# Joint contributions of preview and task instructions on visual search strategy selection

**DOI:** 10.3758/s13414-024-02870-1

**Published:** 2024-04-24

**Authors:** Tianyu Zhang, Jessica L. Irons, Heather A. Hansen, Andrew B. Leber

**Affiliations:** 1 Department of Psychology, The Ohio State University, 225 Psychology Building, 1835 Neil Avenue, Columbus, OH 43210 USA; 2 Data61, CSIRO, Eveleigh, Australia

**Keywords:** Attentional control, Visual search, Strategy, Individual differences

## Abstract

People tend to employ suboptimal attention control strategies during visual search. Here we question why people are suboptimal, specifically investigating how knowledge of the optimal strategies and the time available to apply such strategies affect strategy use. We used the Adaptive Choice Visual Search (ACVS), a task designed to assess attentional control optimality. We used explicit strategy instructions to manipulate explicit strategy knowledge, and we used display previews to manipulate time to apply the strategies. In the first two experiments, the strategy instructions increased optimality. However, the preview manipulation did not significantly boost optimality for participants who did not receive strategy instruction. Finally, in Experiments 3A and 3B, we jointly manipulated preview and instruction with a larger sample size. Preview and instruction both produced significant main effects; furthermore, they interacted significantly, such that the beneficial effect of instructions emerged with greater preview time. Taken together, these results have important implications for understanding the strategic use of attentional control. Individuals with explicit knowledge of the optimal strategy are more likely to exploit relevant information in their visual environment, but only to the extent that they have the time to do so.

**Supplementary information:**

The online version contains supplementary material available at 10.3758/s13414-024-02870-1.

## Introduction

The attentional control system is a powerful, adaptable mechanism critical for many everyday activities. It allows us to manipulate in a goal-directed manner which of the many sources of incoming information are prioritized and which are ignored. Information sources can be prioritized on the basis of a specific property of the desired target, such as a spatial location (Eriksen & Hoffman, 1972; Sperling, 1960), a feature (e.g., color, orientation, or form; Green & Anderson, 1956; Posner et al., 1978; von Wright, 1970), or possibly even a more complex conjunction of features or category label (Bacon & Egeth, 1997). But the extent to which attentional control will benefit performance depends critically on how and when it is wielded. For example, biasing attention towards the color red may be effective when you are searching for your red car in a parking lot filled with cars that are heterogeneous in color, but it will be less effective when searching for your child in a red soccer jersey surrounded by similarly dressed teammates.

To be effective, then, attentional control must be strategic, tailored to the current environment and task demands. But do people use attentional control in a strategic manner? Surprisingly, evidence suggests that observers frequently use attentional control suboptimally (Bacon & Egeth, 1994). More recently, several studies have begun to identify and characterize a variety of ways in which attentional control is used suboptimally (Boot et al., 2009; Clarke et al., 2022; Irons & Leber, 2016; Nowakowska et al., 2017; Rajsic et al., 2015).

Our lab created the Adaptive Choice Visual Search (ACVS) task to systematically investigate attentional strategy use (Irons & Leber, 2016, 2018). In what we currently use as the “standard” version of the task (Irons & Leber, 2018; Exp. 2), observers view a search display of red, blue, and green squares embedded with two targets: one red and one blue square, each containing a digit within a pre-specified range (2–5). Participants can choose to search for either target on each trial, as only one target needs to be reported. Under such conditions, observers typically use subset search, in which they search through only one color-based set of items (either red or blue) for the target. To manipulate the utility of search for each target, we manipulate the ratio of red to blue items from trial to trial. On some trials, there are roughly twice as many red squares as blue squares. In this case, searching through the blue squares to find the blue target takes less time than searching through the red squares for the red target, making blue the optimal target choice. On other trials, there are about twice as many blue squares as red squares, making red optimal. Therefore, our main measure of strategy use is the percent of optimal choices (optimality), or the percent of trials in which individuals choose the target from the smallest subset. The key finding from the ACVS task is that strategy choice is far from optimal, typically not exceeding 70%, although there is substantial individual variation in this number (see Irons & Leber, 2020).

Why is performance so suboptimal? To address this question, it is important to consider that maintaining optimal attentional control in these tasks requires multiple, interconnected steps. The individual must, for example, appraise the environment (Hansen et al., 2019), determine what the candidate attentional control settings are, evaluate the effectiveness of each setting within the current environment (Cain et al., 2012; O’Leary & Sloutsky, 2017; Wolfe, 2013), and finally decide upon the best option, all before search is commenced. Failing to perform any of these component steps adequately may result in suboptimal performance. We have previously posited that selection of a suboptimal strategy may be due to an unwillingness to engage in these component steps because of the effort required to do so (Irons & Leber, 2018, 2020; see also Kristjánsson et al., 2018). After all, sustained proactive control and maintenance of task goals is cognitively demanding (Braver et al., 2007; Chatham et al., 2009; Locke & Braver, 2008). Employing conflict-monitoring mechanisms is likewise resource demanding (Botvinick et al., 2001; Lorist et al., 2005), and switching cognitive strategies is effortful (Arrington, & Logan, 2004; Kool et al., 2010). If an observer is not willing to exert the effort to undertake all of these steps, they may adopt a less effective but less demanding strategy, abiding by the principle of satisficing (Simon, 1956; Yu et al., 2023). For example, in the ACVS task, participants may choose the same target color on every trial, or select a target color at random, because doing so is less effortful than using the optimal strategy.

However, effort may not be the only variable governing strategy choice. In this paper we investigate the role of two additional variables: time and knowledge. First, what if observers are willing to expend the effort required to achieve the optimal strategy but do not have sufficient time to do so? Appraising the visual environment, evaluating different possible strategies, choosing the best one, and implementing the chosen strategy are all time-consuming steps (Wolfe et al., 2004). Even though participants may benefit from faster task performance by choosing the optimal strategy, they must invest time executing the steps required to do so. Instead of investing this time while the search array is available, participants may either believe it is faster to begin searching right away or simply prefer to do so. To test this possibility, we manipulate a display preview, which provides the color subset information to the participants prior to the onset of the target and distractor digits. During this preview time, participants who take on the cognitive steps required to choose and implement the optimal strategy can do so without having to give up the opportunity to begin searching as soon as the display items appear. We have some reason to expect that the availability of the preview display will boost optimality, based on the findings of Hansen et al. (2019). In that study, we presented participants with a preview on all trials and manipulated the presence of a secondary line-judgment task during the preview period, to vary participants’ opportunity to appraise the displays and configure the optimal strategy. The inclusion of the line-judgment task during the preview period – which disrupted the participants’ use of the preview – worsened optimality. Subsequent studies have included previews on the theoretical assumption that they boost optimality (Li et al., 2022; McKinney et al., 2022). Here, we systematically manipulate preview to directly establish the relationship between appraisal time and optimality. Note that in this paper, we refer to a “preview benefit” as an increase in optimality associated with viewing display previews. Our use of this term differs from studies of visual marking, in which previewing a set of nontarget items in a search display allows observers to ignore these items once the full search array appears, thus facilitating performance (see Watson & Humphreys, 1997).

Second, what if observers are willing to expend effort but do not know the optimal strategy? These individuals may have an incomplete mental model or understanding of the task, and therefore cannot fully execute the component steps required to perform optimally. In this paper, we endow participants with knowledge of the optimal strategy and measure how this explicit instructional manipulation impacts strategy use. Previous studies – including those from our lab – have already employed an instructional manipulation to boost strategy use, based on the theoretical assumption that it would help (Hansen et al., 2019; Kim et al., 2021; Li et al., 2022; McKinney et al., 2022). However, in this paper, we systematically manipulate instructions to estimate just how much of a boost the knowledge can provide.

The present paper summarizes four experiments that detail the sequence of our investigation into the roles of both time and knowledge. When we began, we had only considered the role of preview without considering instruction. Thus, Experiment 1 consists of a preview manipulation without providing any explicit instructions. To foreshadow, we found a numerical but nonsignificant effect of preview, which led us to investigate further. Experiment 2 consists of an instructional manipulation without providing a preview. To foreshadow this result, we found a robust effect of instructions, but optimality remained well below ceiling.

Following these first two experiments, we began to consider a possible interactive effect between both preview and instruction. While we failed to find a significant effect of preview in Experiment 1, affording participants more time to use the optimal strategy may only work insofar as participants know what the optimal strategy is. We thus predicted that benefits of preview would be greater for participants who receive instruction. Experiments 3A and 3B test and produce support for this hypothesis.

### Experiment 1: Preview manipulation (without instruction)

Given that assessment of the color make-up is integral to performing optimally in this task, in Experiment 1 we examined whether observers are more likely to adopt an optimal strategy if they have more opportunity to appraise the search display before beginning the search process. In our previous studies (Irons & Leber, 2016, 2018), the full search display containing all colored squares and digits was presented at once, and participants were asked to search as quickly as possible. Such an abrupt presentation may act as a trigger to launch into serially searching through the display, without first appraising the display. Many individuals adopt “active” strategies for search that involve frequent and fast eye-movements, despite the fact that these strategies are often less effective than strategies that may be more responsive to properties of the search environment (e.g., Boot et al., 2009). If participants have more time to freely appraise the properties of the environment, without the pressure to begin search, they may make more optimal choices.

Rajsic et al. (2015) presented evidence that previewing display information reduced a suboptimal “confirmation bias” in visual search. They asked participants to determine whether a specific target letter was in a cued color set or not. The optimal way to perform the task would be to search the smaller set of items and then determine the correct response based on the color cue. However, participants tended to search through the cued color, whether or not it contained greater or fewer items than the uncued color. This confirmation bias, however, was reduced when the color information was previewed. There are several notable differences between the task of Rajsic et al. and the ACVS. The ACVS does not include changes in target instructions from trial to trial, and it is designed to measure strategy more directly via a target choice variable. Moreover, how individuals optimize strategy tends to differ widely across distinct attentional tasks (Clarke et al., 2022). Nevertheless, the study by Rajsic et al. provides initial evidence that appraisal time can be used by participants to improve their attentional strategies.

Participants in Experiment 1 completed two conditions of the search task. In the baseline condition, the full search display was presented following a blank intertrial interval (ITI). In the preview condition, the search display was presented without any digits for 1 s prior to the presentation of the full search display. This gave participants extra time to assess the search display composition and decide upon the optimal target color before the actual search began. Note that the preview manipulation is a passive approach to improving strategy use; it is still up to the individual to decide to engage in the appraisal process. Here, we focused on the percent of optimal choices as the measure of strategy usage. If individuals use the preview time to assess the display and engage the optimal strategy, then their rate of optimal choices should be higher in preview blocks than in non-preview blocks.

### Method

#### Pre-registration

Methods and analyses for Experiment 1 were pre-registered prior to data collection on the Open Science Framework website (https://osf.io/frgp6).

#### Participants

Twenty-four young adults (17 men and seven women; age range 18–22 years, *M* = 19.54 years) recruited at The Ohio State University participated in return for psychology course credit. For this first experiment, we sought a design that would be sensitive to an effect size of about *d* = 0. 7. Our chosen sample size was sufficient to provide power of > .90. All procedures were approved by The Ohio State University Internal Review Board (IRB).

#### Stimuli and materials

**Fig. 1:**
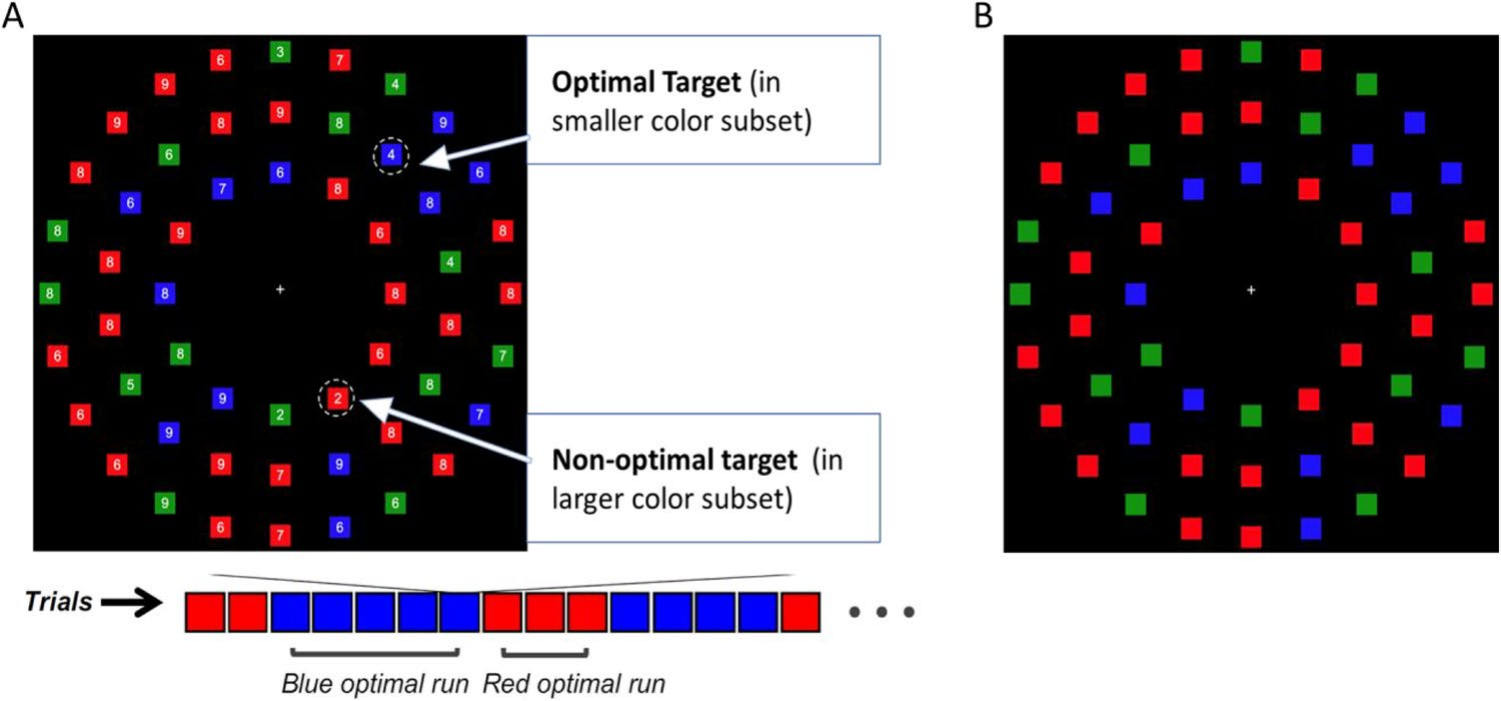
(**A**) An example search trial with targets circled for illustration (note circles did not appear in the experiment). In this example, the blue square containing the target number 4 is considered more optimal to find than the red square containing the target number 2 because there are half as many blue squares to search through as there are red squares. A sample trial order is presented below, where the optimal color changes in runs of one to six trials. (**B**) An example of the preview of the search display with colored squares only

Search displays were composed of 54 small squares (1° x 1°) evenly spaced in three concentric rings around fixation (12 squares in the inner ring, 18 in the middle ring, and 24 in the outer ring). The inner ring was at 6.3° eccentricity, the middle ring was at 9.4° eccentricity, and the outer ring was at 12.4° eccentricity from a central fixation cross. Each square was colored red, blue, or green. Half of the trials contained “red optimal” displays and half contained “blue optimal” displays. Red optimal displays were composed of 13 red, 27 blue, and 14 green squares, and blue optimal displays contained 27 red, 13 blue, and 14 green squares. The spatial locations of the different colored squares were randomized on each trial. Short runs of red optimal and short runs of blue optimal trials were interspersed within a block (see Fig. 1A). The length of the runs was randomized between one and six trials, with the restriction that there was an equal number of each run length for each display type within the block.

The preview stage consisted of the search display with colored squares only (see Fig. 1B). In full search, a white digit between 2 and 9 (0.48°, font: Arial) was presented on each square. Two targets were embedded in every display: a red square and a blue square each containing a digit between 2 and 5 inclusive. The two targets always contained different digits, to distinguish responses to the two targets. All other red and blue squares (distractors) had a digit between 6 and 9. Green squares could contain any digit between 2 and 9, to ensure participants did not ignore color in favor of a digit-only search.

#### Procedure

Participants were informed that each search display would contain two targets, with one in a red square and one in a blue square, but they needed to only find one on each trial. They were always free to search for either target on each trial.^1^ Upon finding a target, participants identified the digit inside the target by pressing the corresponding key on the keyboard (V, B, N, or M corresponding to 2, 3, 4, or 5, respectively). No information was provided about the preview manipulation or the optimal strategy for search.

Participants completed ten practice trials of the baseline condition (no preview display), followed by six experimental blocks of 84 trials. Of these six blocks, three blocks were baseline search and three were preview search. Baseline and preview blocks were interspersed, and the condition of the first block was counterbalanced across participants. In baseline search blocks, the trial sequence consisted of a central fixation cross for 2 s (ITI), followed by the full search display, which remained on-screen until a response was made. In the preview blocks, the ITI was presented for 1 s, followed by the preview (colored squares without digits) for 1 s, and then the full search display (colored squares and digits) until response. Target identity and spatial location, and distractor identity and spatial location, were randomized across trials.

### Results and discussion

Response accuracy (i.e., correctly identifying either of the two targets) was at ceiling for all participants (*M* = 98.58%) and did not vary between baseline and preview conditions, *t*(23) = .74, *p* = .47, *d* = 0.21. Response time (RT) was significantly faster in the preview search blocks (*M* = 2798ms) than on baseline blocks (*M* = 3242ms, *t*(23) = 8.23, *p* < .001, *d* = 1.69). Furthermore, across individuals, RT and optimal choices were significantly correlated (*r* = -.52, *t*(22) = 8.75, *p* = .007), indicating that optimal choice rate is associated with faster performance.

**Fig. 2:**
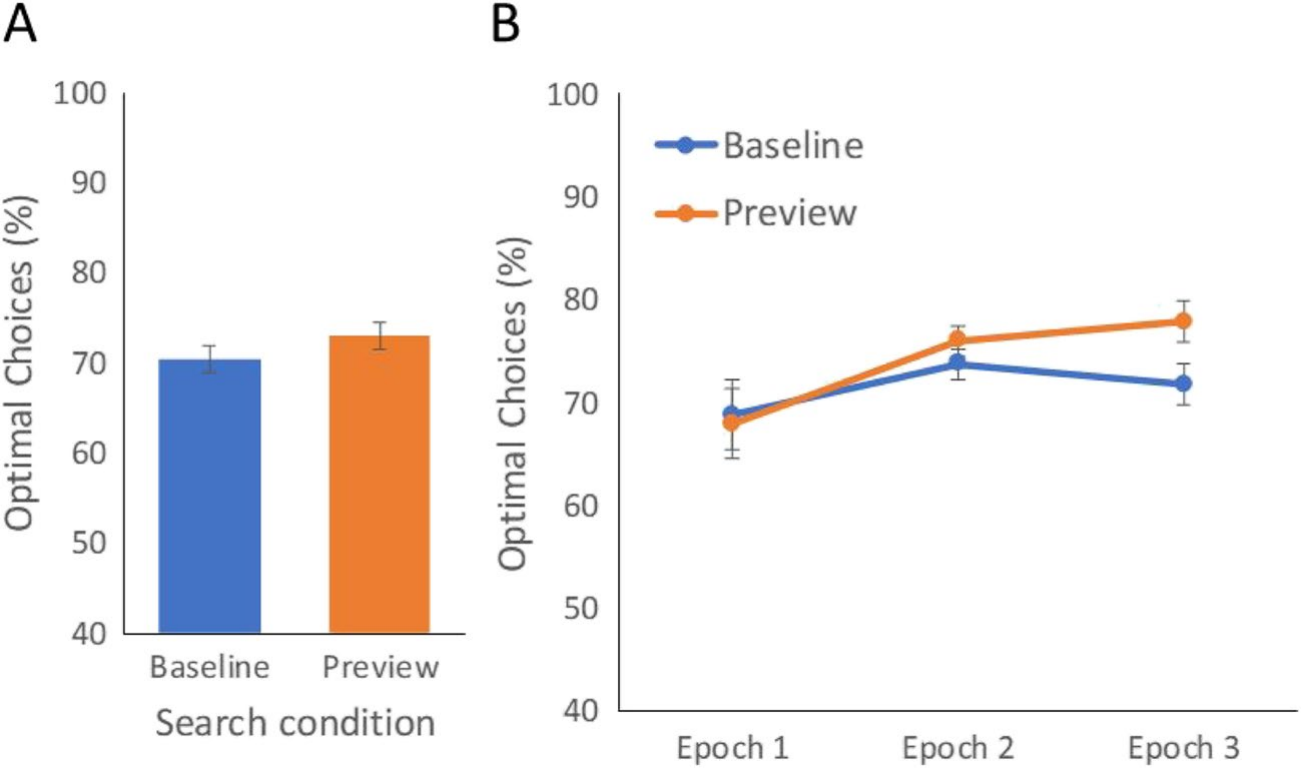
Experiment 1: Percent of optimal choices (**A**) across search conditions and (**B**) across three epochs, which each contained two blocks, or 168 trials. Error bars indicate the standard error of the mean differences across search conditions

Overall, the rate of optimal choices was 71.65%, which is well below optimal. Averaged across the entire experiment, the percent of optimal choices was not significantly higher on preview blocks (72.97%) than on baseline blocks (70.34%), *t*(23) = 1.79, *p* = .087, *d* = 0.37 (Fig. 2A).

While there was no evidence that a preview benefits optimal choices when trials are combined across the entire experiment, it is possible that a preview effect was present at the start of the experiment and later obscured by learning. Through experience with the preview condition, participants may have learned that appraising the display was beneficial and carried this strategy over to the baseline blocks. Alternatively, perhaps participants would get better at incorporating the optimal strategy over time. We thus did an exploratory analysis in which we separated the preview and baseline data into three epochs each. A two-way ANOVA found a significant main effect of epoch, *F*(2,46) = 6.34, *p* = .004, ${\eta }_{p}^{2}$= .021 (Fig. 2B). However, the interaction between preview condition and epoch did not reach significance, *F*(2,46) = 2.09, *p* = .134, ${\eta }_{p}^{2}$= .005. Nevertheless, numerically, it seemed that a preview effect emerged later in the experiment.^2^

Experiment 1 showed that, overall, providing participants with more opportunity to appraise the search display and make a decision about target color provided little improvement to strategy use. However, it is possible that some benefit for preview was beginning to emerge later in the experiment. If such an effect is real, we can speculate that it emerges late because participants need time to learn *how* to use the preview. Note that overall percent of optimal choices in the first epoch was somewhat lower than the remainder of the experiment for both preview and baseline blocks, which suggests that some incremental learning of the optimal strategy was occurring in both conditions over time. Once this learning had been developed, participants may have been more able to take advantage of the extra preview time to appraise the display.

## Experiment 2: Instruction manipulation (without preview)

In Experiment 2, we shift gears and test the hypothesis that being made explicitly aware of the optimal strategy increases its usage. Two groups of participants performed a baseline version of the ACVS (without a preview phase). Prior to beginning the experiment, one group (the “instruction” group) was given additional instructions informing them of the optimal strategy. Specifically, they were told that there would be unequal numbers of red and blue squares in each display, and that searching in the color with the fewest squares has been observed to produce the best performance. If suboptimal performance is driven by a lack of awareness of the optimal strategy, the Instruction group should perform more optimally than the Control group.

### Method

#### Participants

Forty-nine individuals participated in return for course credit (19 men and 30 women; age range 18–24 years, *M* = 18.7 years). Data from one participant, whose accuracy was more than 3 SDs below the other participants, were excluded. Participants were randomly assigned to either the Instruction group or the Control group (*N* = 24 per group). Given that our manipulation was between-subjects and likely requires a greater sample size to obtain sufficient power, compared to the within-subjects manipulations in Experiment 1, we elected to double the sample size used previously. Such a sample size was sufficient to provide a power of > .80 to find a moderate to large effect size (*d* = 0.75).

#### Stimuli and procedure

The task and stimuli were identical to the baseline search blocks in Experiment 1. No preview of the display was given. All participants completed ten practice trials followed by four experimental blocks of 84 trials. At the start of the practice, both groups were given standard instructions that a red and blue target would be present on each trial, and that they were always free to search for either one. The practice trials were then administered. Next, the Instruction group received the following additional instructions: “You may have noticed that sometimes there are more red squares in the display, and sometimes more blue squares. Previous experiments have shown that the fastest way to do the task is to look for whichever color has the fewest squares. For example, if there are fewer red than blue squares, it will usually be faster to look for the red target.” The experimenter read the instructions to the participants and then showed them three example displays, pointing out the optimal target color in each display. The Control group did not receive any further instructions or examples. All participants then completed the experimental trials.

After running approximately one-third of our sample, we introduced an exploratory strategy questionnaire to assess people’s knowledge of the optimal strategy. We do not report the results here, but we include a more complete awareness assessment in Experiments 3A and 3B.

### Results

As in the previous experiments, accuracy was at ceiling (*M* = 97.53%) and did not vary across groups, *t*(23) = 1.14, *p* = .26, *d* = 0.34.

**Fig. 3:**
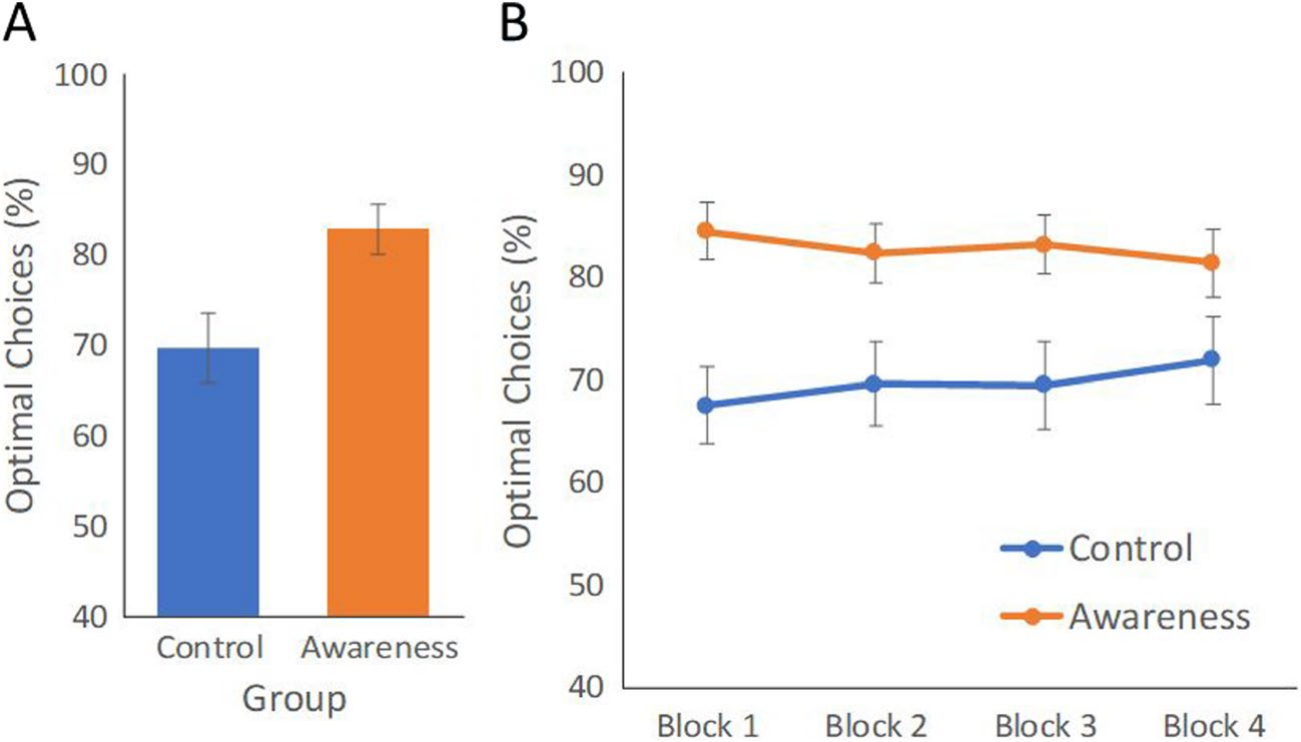
Experiment 2: Percent of optimal choices (**A**) for Control and Instruction groups, and (**B**) across blocks. Error bars indicate standard error of the mean

Analysis of optimal choices indicated that awareness instructions did indeed influence optimal choice rate: participants in the Instruction group (*M* = 82.83%) were significantly more optimal than participants in the Control group (*M* = 69.62%), *t*(23) = 2.81, *p* = .007, *d* = 0.82 (Fig. 3A). The difference in RTs across the two groups was not significant (Instruction group *M* = 2,975 ms; Control group *M* = 3,175 ms; *t*(23) = 1.29, *p* = .20, *d* = 0.37). However, over the entire sample, combined RTs and optimal choices were significantly correlated (*r* = -.51, *t*(46) = 4.02, *p* < .001). For completeness, we also calculated the correlation within each group (Instruction group: *r* = .63, *t*(22) = 3.84, *p* < .001 and Control group: *r* = .39, *t*(22) = .201, *p* = .056)^3^, once again demonstrating a performance benefit for searching the smaller colored subset.

To examine whether the difference between groups changed across time, we assessed optimal choices across the four experiment blocks. Optimal choices did not vary significantly across blocks, *F*(3, 138) = .10*, p* = .96*,* ${\eta }_{p}^{2}$ = .002, nor was there any interaction between block and group, *F*(3, 138) = 2.05*, p* = .11*,* ${\eta }_{p}^{2}$ =.043 (Fig. 3B).

Overall, these results are consistent with the view that awareness supports optimal strategy use. Optimal choices were much higher for individuals who were instructed on the optimal strategy. Thus, instruction of the optimal strategy provides a workable method to promote the use of the optimal strategy.

## Experiment 3A: Joint preview and instruction manipulation

Experiment 2 demonstrated that instructing participants on the optimal strategy increases optimal performance. However, despite complete knowledge of the task, participants in the Instruction group were still not fully optimal, and individual optimality rates within this group varied from 35.9% to 96.7%. Thus, there are clearly other factors affecting strategy choice. Even if individuals know what the optimal strategy is, if they lack the time or ability to engage in all of the component steps of implementing an optimal strategy, they may still perform suboptimally. Here we question whether we can maximize optimal performance by both informing participants of the optimal strategy *and* giving them a preview period to take advantage of it. In this experiment, we combined the interventions investigated in Experiment 1 and Experiment 2. Our data from Experiment 1 suggested that previewing the display may have improved optimal choices only later in the experiment, although we did not obtain statistical support for a preview benefit. Nevertheless, we speculated that observers may need to learn the optimal strategy before they can make use of the preview. In other words, the preview benefit might be dependent on awareness. In Experiment 3A, we tested this hypothesis by examining whether a preview period increases optimal choices when participants are made fully aware of the optimal strategy.

In designing this experiment, we considered several issues. First, our failure to detect a significant preview effect in Experiment 1 may have been due to insufficient power. If the observed effect size represents the true effect (*d* =.37), we would have needed a sample of 60 participants to obtain 80% power to detect this effect. Thus, we increased the sample size in Experiment 3A. Second, the duration of the preview in Experiment 1 was only fixed to 1 s. We have theorized that 1-s previews benefited performance in Hansen et al. (2019); it is possible that this duration was not quite long enough to allow participants to fully implement the optimal strategy prior to the onset of the search items. Thus, in Experiment 3A, we included preview durations ranging from 0 ms (i.e., no preview) through 2,000 ms. Third, the no-preview control condition in Experiment 1 was arguably not perfectly matched to the preview condition; that is, the preview trials introduced a full array of squares, which were not present in the control trials. For better experimental control, Experiment 3A matched the trials by providing gray placeholders when the color preview was not being presented.

### Method

#### Pre-registration

Methods and analyses for Experiment 3A were pre-registered prior to data collection on the Open Science Framework website (https://osf.io/3qdnz). All analyses not in the preregistration plan are declared as exploratory.

#### Participants

In this experiment, the sample size was set at 120 participants. An additional ten participants were tested but were excluded due to poor accuracy (more than 3 SDs below the group mean). The final sample consisted of 70 men, 48 women, one non-binary person, and one unreported (age *M* = 30, *SD* = 6.3). Participants were randomly assigned to either an Instruction group (with optimal strategy instruction, *N* = 60) or a Control group (without optimal strategy instruction, *N* = 60). Data collection was shifted to online due to the COVID-19 pandemic. Participants were recruited through Prolific (Prolific.co) with a minimum approval rate of 96% and a minimum number of submissions of 50. Participants were compensated for their time at a rate of $10/h.

To study the effect of preview, a sample size of 60 provides power of 0.8 to detect the effect of preview on optimality when there is no instruction of the optimal strategy (using *d* = 0.37, based on the Experiment 1 data). Also, we estimated power of 0.99 to detect the preview effect when optimal strategy instructions are presented (*d* = 1.39, based on unpublished pilot data). To study the main effect of instructions, we estimated that a sample size of 60 in each group would give us power of 0.99 to detect the effect (based on the effect size obtained from Experiment 2, *d* = .82). To study the interaction between instructions and preview, we estimated that a sample of 120 (60 per group) would give us a power of > 0.999 to detect a large interaction effect; this was based on a cross-experiment comparison between Experiment 1 and unpublished pilot data, which yielded ${\eta }_{p}^{2}$ = 0.134.

#### Stimuli and procedure

The stimuli were similar to Experiments 1 and 2. Since this experiment was conducted online, stimuli were presented on participants’ own devices with variable sizes and refresh rates. We chose to create the stimuli in such a way that, with a standard 24-in. display and a viewing distance of 60 cm, the visual angles would be the same as reported in Experiment 1.

To better match the stimulus presentation across conditions, we modified the preview stage for all three preview durations (0 ms, 1,000 ms, 2,000 ms). Prior to presenting the search array (including digits ranging from 1–9), we presented square placeholder items at each location for 2,000 ms. In the 0-ms preview condition (no color preview), these squares were all gray until the moment the search array appeared. In the 1,000-ms preview, the placeholders were gray for the first 1,000 ms, and then their search-task colors were revealed for 1,000 ms. Finally, in the 2,000-ms preview, the placeholders were shown in their search-task colors for the full 2,000 ms.

As described in our preregistration, we intended to use the same run lengths of the small subset color as we did for Experiments 1 and 2 (i.e., equally sampling run lengths of 1–6). However, due to coding error, the run lengths were actually sampled equally from 1 to 5. This effectively changed the mean run length from 3.5 to 3.0. We do not expect this change to have a noticeable impact on our results, as we have previously shown that run length does not significantly affect optimality rates (Shaw et al., 2020).

Participants performed all three preview conditions in six separate blocks, with the first three blocks of 84 trials fully counterbalanced across subjects. The second set of three blocks repeated the ordering of the first three blocks. At the start of the experiment, both groups were given standard instructions, and the Instruction group received the additional instructions from Experiment 2, informing them of the optimal strategy.

At the end of the task, participants completed a “strategy awareness check,” designed to check whether participants were aware of the association between the number of red/blue distractors and performance. This comprised ten displays without digits (similar to the preview displays). Participants were asked to imagine that they were performing the search task, and for each display, indicate whether they thought it would be faster to search for the red target or the blue target. Responses were made by clicking on of two boxes marked “red” and “blue” in the center of the display.

### Results

Accuracy was similar across the Instruction (*M* = 97.5%) and Control (M = 96.9%) groups, *t*(118) = 1.29*, p* = .10. RTs were faster in the Instruction group (*M* = 2,504 ms) than in the Control group (*M* = 2,687 ms), *t*(118) = 1.69*, p* = .047. RTs were significantly correlated with optimality (*r* = -.57, *t*(118) = 57.76, *p* < .001) over the entire sample (Instruction group: *r* = .52, *t*(58) = 4.63, *p* < .001 and Control group: *r* = .61, *t*(58) = 5.86, *p* < .001).

#### Optimality

**Fig. 4:**
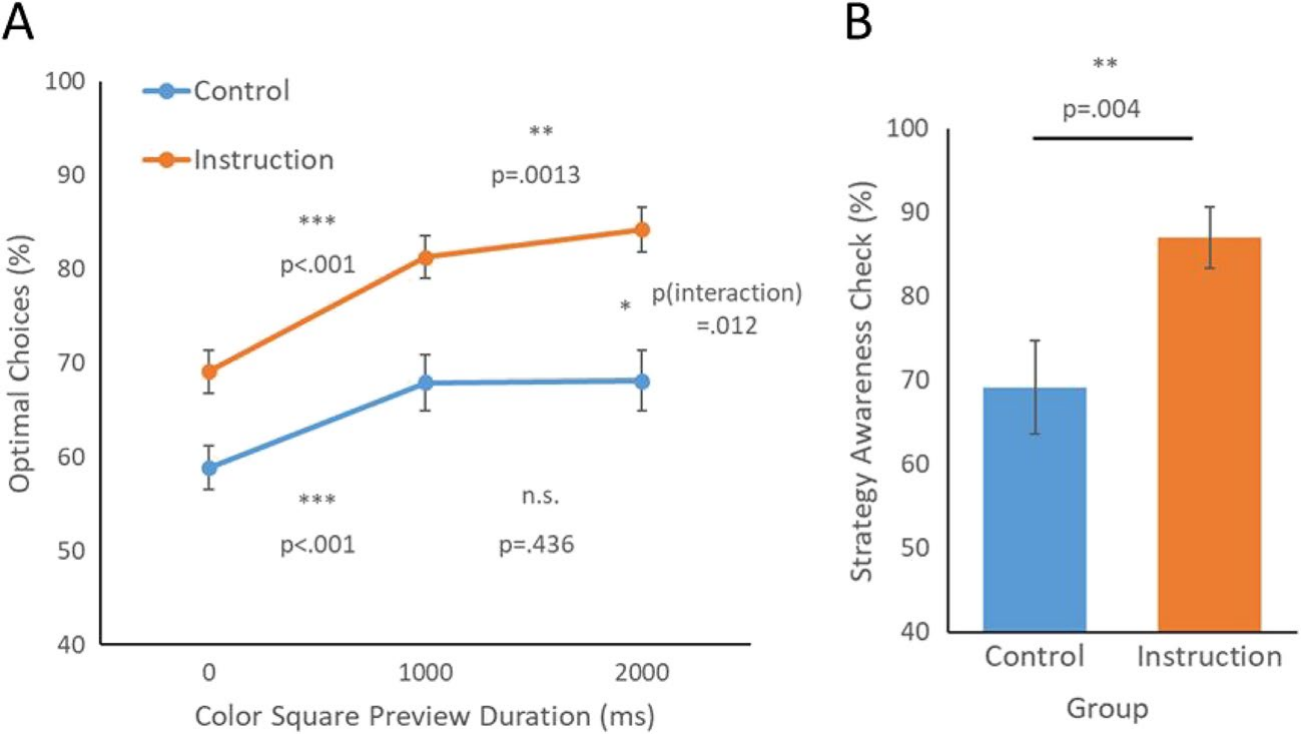
Experiment 3A. (**A**) Percent of optimal choices for Control and Instruction groups across three preview conditions. (**B**) Percent of optimal choices on the strategy awareness check in Experiment 3A. Error bars indicate standard error of the mean

A 3 (Preview condition: 0 s, 1 s, 2 s) × 2 (Group: Instruction vs. Control) mixed ANOVA was applied to optimality rate. Results revealed a main effect of Instruction group (*F*(1,118) = 14.39*, p* < .001*,* ${\eta }_{p}^{2}$ = .109) and a main effect of preview (*F*(2,236) = 91.14*, p* < .001*,* ${\eta }_{p}^{2}$ = .435) (Fig. 4A). Furthermore, we found a significant interaction between these two factors, *F*(2,236) = 4.54*, p* = .012*,* ${\eta }_{p}^{2}$ = .037, indicating that the preview effect was more robust when instruction of the optimal strategy was provided. T-tests showed that, for the Instruction group, optimality was significantly higher with a 1-s preview (*M* = .813) than a 0-s preview condition (*M* = .691), *t*(59) = 9.08*, p* < .001*, d* = 1.17. Further, optimality in this group was greater for the 2-s than the 1-s preview (*M* = .842), *t*(59) = 3.15*, p* = .001*, d* = 0.407.

Additional t-tests for the Control group clarified the effect of preview. Here, without an instruction of the optimal strategy, participants did show greater optimality in a 1-s preview condition (*M* = .679) than the 0-s preview condition (*M* = .589), *t*(59) = 5.92*, p* < .001*, d* = 0.764. However, the longer duration 2-s preview did not further improve optimality beyond the 1-s preview (*M* = .681), *t*(59) = .155*, p* = .439*, d* = 0.02.

Finally, we assessed the results of the strategy awareness check. As expected, the Instruction group chose the optimal task color more frequently than the Control group (Instruction *M* = 87.00%; Control *M* = 69.16%; *t*(118) = 2.70, *p* = .004, *d* = 0.49; see Fig. 4B). Across all participants, optimal choices in the strategy awareness check were significantly correlated with optimal choices in the ACVS (*r* = .37, *p* < .001).

## Experiment 3B: Joint manipulation with additional preview durations

Experiment 3A clarified the joint effects of both preview and instruction. Interestingly, while we failed to find a significant preview effect without instruction in Experiment 1, the higher-powered manipulation – via greater sample size – in Experiment 3A did yield a significant result. Thus, preview can work even without instruction. But, how long a preview is sufficient to boost optimality? Prior work has shown that participants have higher optimality when there is at least some available time to appraise the display, such as when a concurrent task during a 1-s preview is completed with time to spare (Hansen et al., 2019). To better characterize the time course of preview effects, we decided to use the same paradigm as in Experiment 3A and investigate three new durations (250 ms, 500 ms, and 750 ms).

### Method

#### Participants

Similar to Experiment 3A, the sample size of Experiment 3B was set at 120 participants in total with 60 participants in each group (Instruction or Control). An additional 21 participants were tested but were excluded due to poor accuracy (more than 3 SDs below the group mean or lower than .80). The final sample consisted of 74 men, 42 women, three non-binary individuals, and one unreported (age *M* = 30 years, *SD* = 5.7 years).

#### Stimuli and procedure

The task and stimuli were identical to Experiment 3A. Participants performed all three preview conditions (250 ms, 500 ms, and 750 ms) in separate counterbalanced blocks. The standard instructions were provided to both groups and the additional instructions regarding the optimal strategy were given to the Instruction group. As in Experiment 3A, participants also completed the strategy awareness check.

### Results

As reported in Experiment 3A, accuracy was similar across Instruction (*M* = 96.0%) and Control (*M* = 95.8%) groups, *t*(118) = .257*, p* = .399. Interestingly, RTs were similar in both the Instruction group (*M* = 2,621 ms) and the Control group (*M* = 2,580 ms), *t*(118) = .362*, p* = .36.^4^ However, the significant negative correlation between RTs and optimality remained (*r* = -.56, *t*(118) = 53.31, *p* < .001; Instruction group: *r* = .69, *t*(58) = 7.32, *p* < .001 and Control group: *r* = .50, *t*(58) = 4.43, *p* < .001).

**Fig. 5:**
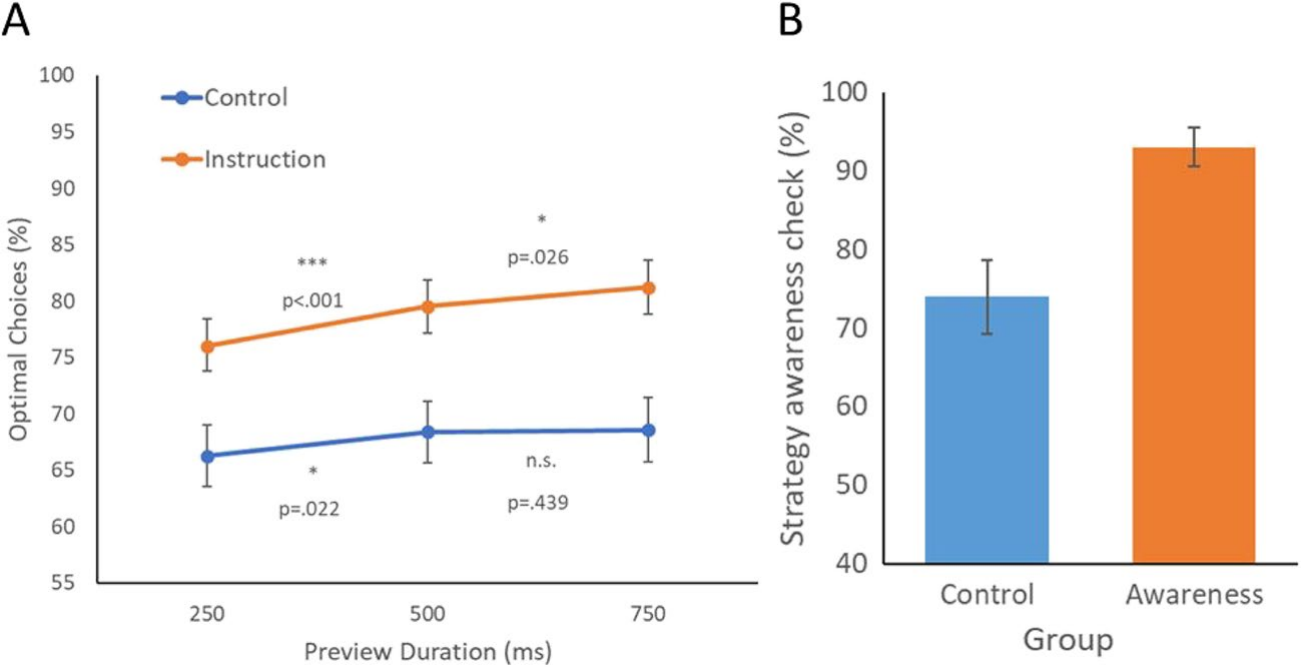
Experiment 3B. (**A**) Percent of optimal choices for Control and Instruction groups across three preview conditions. (**B**) Percent of optimal choices on the strategy awareness check in Experiment 3A. Error bars indicate standard error of the mean

A similar 3 (condition: 250 ms, 500 ms, 750 ms) × 2 (group: instruction vs. control) mixed ANOVA was applied on optimality rate. Results revealed a main effect of Instruction group (*F*(1,118) = 9.85*, p* =.002*,* ${\eta }_{p}^{2}$ = .078) and a main effect of preview (*F*(2,236) = 14.73*, p* < .001*,* ${\eta }_{p}^{2}$ = .111) (Fig. 5A). However, the interaction was not significant here *F*(2,236) = 2.05*, p* = .13*,* ${\eta }_{p}^{2}$ = .017. Additional t-tests revealed that optimality was significantly higher with a 500-ms preview (*M* = .795) than a 250-ms preview (*M* = .760*), t*(59) = 4.55*, p* < .001*, d* = 0.588. A further improvement was observed with a 750-ms preview compared to 500-ms preview (*M* =.812*), t*(59) = 1.97*, p* = .026*, d* = 0.255. In contrast, in the Control group, optimality was first increased from 250 ms to 500 ms of preview (*t*(59) = 2.06*, p* = .022*, d* = 0.266) but failed to improve further from 500 ms to 750 ms (*t*(59) = .153*, p* = .439*, d* = 0.019). The results of the strategy awareness check replicated the findings of Experiment 3A, which revealed that instruction improved participants’ awareness of the optimal strategy (Instruction *M* = 93.00%; Control *M* = 74.00%; *t*(118) = 3.56, *p* < .001, *d* = 0.65; see Fig. 5B).

**Fig. 6:**
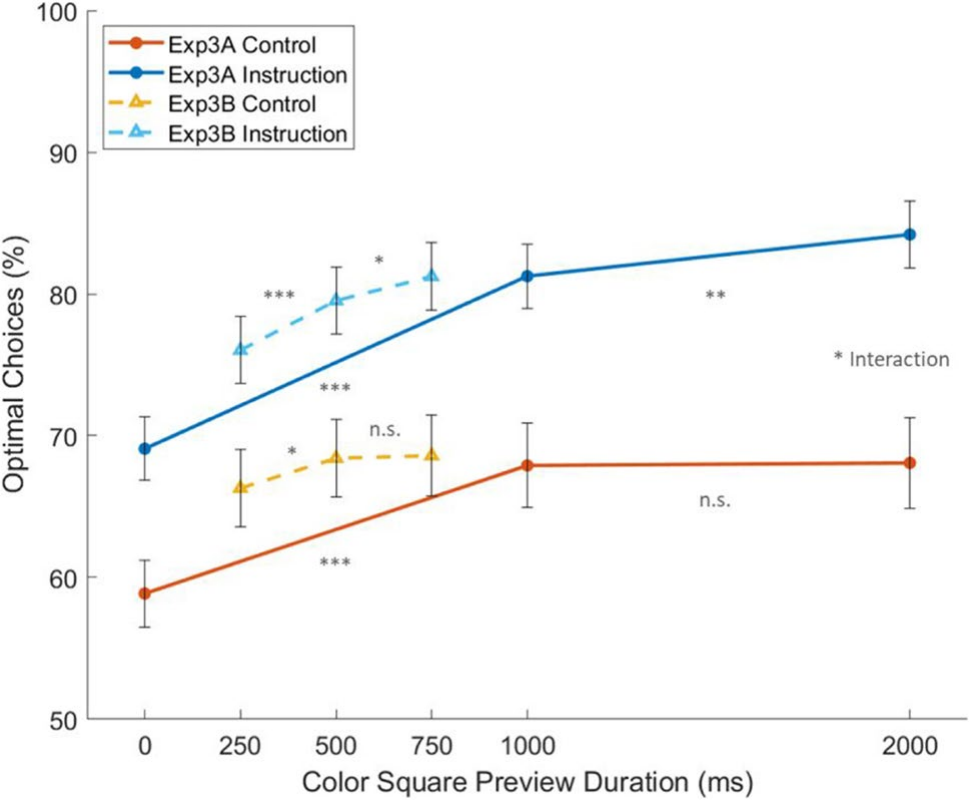
Combined data for the percent of optimal choices across conditions

By combining the data from Experiments 3A and 3B (Fig. 6), we could see clearly that, if the knowledge of the optimal strategy was provided, a longer duration of preview continued to benefit optimality. However, in the Control group, the preview effect plateaued by 500 ms.

### Discussion

People sometimes settle for nonoptimal visual search strategies (Bacon & Egeth, 1994; Irons & Leber, 2016; Nowakowska et al., 2017; Rajsic et al., 2015; Yu et al., 2023). In seeking to advance our understanding of why this is the case, we questioned the contributing roles of explicit knowledge and time to appraise the environment, using a modified version of the ACVS (Irons & Leber, 2018). Overall, we found joint and interactive contributions of explicit strategy knowledge and appraisal time.^5^

In Experiment 1, we first manipulated the preview time in a group without explicit instructions, and we found a numerical but nonsignificant boost in optimality due to the preview. In Experiment 2, we manipulated instructions without a preview, and we found a significant effect of strategy knowledge. We then speculated that preview effects would be most robust in the presence of explicit instructions, which led us to jointly manipulate both factors while also boosting sample size to increase power (Experiments 3A and 3B). We found main effects of both preview and instruction, and we confirmed the speculation that the two indeed interact.

Overall, the results of this study suggest multiple barriers to optimal performance in visual search: appraisal time and explicit knowledge. We found that each of these variables can explain suboptimal behavior. Moreover, they interact, such that the greatest impact of explicit knowledge is realized with longer appraisal time. Thus, for observers to demonstrate maximum levels of optimality, it would seem that multiple conditions must be satisfied.

We must take care to emphasize that sufficient appraisal time and lack of relevant knowledge cannot be the only factors preventing people from being optimal. As we can tell from Experiments 3A and 3B, for those participants who showed clear evidence of optimal strategy knowledge, their optimality did not reach ceiling, even at the longest preview durations. We believe that factors discussed in the *Introduction*, such as avoidance of cognitive effort (Irons & Leber, 2018) and switching between attentional control settings, may also influence people’s visual search behaviors. In fact, one might interpret the preview benefit observed here as consistent with an effort account. That is, in the absence of a preview, individuals may find it more effortful or subjectively unpleasant to take time to appraise the display and choose the optimal target color, since any delays at this stage would delay the commencement of search. Critically, while there is some tradeoff between investing time to appraise the display and commence search during the no-preview condition, it is still objectively optimal to choose the smaller subset in this condition; we have reliably observed a strong relationship between optimality and RT when preview is not present (Irons & Leber, 2018; Li et al., 2022; McKinney et al., 2022).

Beyond the theoretical advances of this work, our parametric manipulation of preview duration in Experiments 3A and 3B helps to provide practical guidance for future experimentation with the ACVS. For individuals seeking a substantial boost in optimality due to preview while minimizing the length of the experiment, we might recommend a preview duration of at least 500 ms.

Relatedly, it is interesting that we found significantly greater optimality at the 2,000- versus 1,000-ms preview duration for the Instruction group in Experiment 3A. It is doubtful that participants need 2,000 ms to perform the task optimally. Specifically, in numerosity discrimination or judgement tasks, the RTs have ranged from 500 ms to 1,000 ms (see Ratcliff et al., 2015; Ratcliff & McKoon, 2018). Moreover, in our previous work in which we required participants to report only the optimal target without any preview (Irons & Leber, 2018), we found ceiling-level accuracy with even shorter RTs, demonstrating that long previews are not necessary to appraise the display and complete any proactive configuration of their attentional templates to focus on the smaller subset. Thus, it is unlikely that the continued improvement of optimality up through the 2-s preview is due to needed processing time. We can speculate instead that such improvement in optimality might be due to the avoidance of boredom or the need to engage in cognitive processing (Cacioppo & Petty, 1982; Wu et al., 2022). Clearly, while previews between 500 and 1,000 ms provided sufficient time to comfortably implement the optimal strategy, some participants – due perhaps to effort avoidance – were still unwilling to do so. In the 2-s preview condition, however, the especially long time of waiting for the start of a search trial may have brought on boredom, which could have overridden the desire to avoid cognitive effort in some. This interpretation is, of course, highly speculative, and more studies are needed to directly investigate the potential role of boredom avoidance in attentional strategy selection. Regardless, this interesting finding further supports the conclusion that factors beyond knowledge and insufficient appraisal time solely drive strategy choice.

One additional consideration is why exactly instructions boost performance. Based on our awareness assessment, we are confident that individuals in the Instruction group were more informed about the optimal strategy than those in the Control group. However, it is possible that the instruction not only informed participants but also brought on a demand characteristic, socially pressuring observers to perform the task in the manner they perceived the experimenter to want (Alekseev et al., 2017; Nichols & Maner, 2008). Simply by providing additional information regarding the optimal strategy in the instructions, participants may feel obligated to use it in the search task. As shown in Experiment 2, the optimality in the Instruction group declined numerically, though not significantly, over time, reflecting a possible waning social impact of the instructions. A similar tendency was observed in Experiments 3A and 3B. Thus, one needs to pay close attention to the content of instructions when teaching participants about the optimal strategy. Extra words may be needed to emphasize the high demand for such a method and the freedom to choose any other strategies. One way that future work could test this hypothesis would be to supply our instructions to participants who are already independently aware of the optimal strategy to determine if it still boosts optimality.

Relating to the results we have just discussed, one might question why the awareness check didn’t reach ceiling in the Instruction group, despite all participants being informed of the optimal strategy. Several factors could contribute to this. Participants may not pay enough attention to the instructions, or they could have forgotten the instructions during the experiment. Additionally, participants might harbor doubts about the experimenter's instructions, opting to explore on their own rather than following the recommended strategy. Some may even develop misconceptions, believing it to be quicker to find the target in the more numerous subset. Indeed, we observed some participants favoring the color of the larger subset.

Given that awareness did not reach 100%, and given that strategy choice could theoretically be limited by awareness, we could arguably compare optimality rates to awareness rates, using the awareness rates as a theoretical ceiling for optimality. If optimality remains lower than the explicit awareness rates, then we can more confidently infer the influence of factors other than explicit knowledge on optimality. Our results align with this theoretical framework. Across all preview conditions, our results did show that participants’ optimality (3A: Control group *M* = 65.0%, Instruction group *M* = 78.2%; 3B: Control group *M* = 67.7%, Instruction group *M* = 78.9%) was significantly lower than the reported awareness (3A: Control group *M* = 69.2%, Instruction group *M* = 87.0%; 3B: Control group *M* = 74.0%, Instruction group *M* = 93.0%) in both Experiment 3A (Control group: Wilcoxon sum rank test, *p* = .002, effect size *r* = 0.399; Instruction group: Wilcoxon sum rank test, *p* < .001, effect size *r* = 0.783) and Experiment 3B (Control group: Wilcoxon sum rank test, *p* = .001, effect size *r* = 0.421; Instruction group: Wilcoxon sum rank test, *p* < .001, effect size *r* = 0.927). However, given the fact that the awareness check only contained ten trials per participant, we are cautious not to overemphasize these results. Also, interpretation of these results is limited by *when* participants – particularly in the Control group – discovered the optimal strategy. We have averaged optimality across the entire session while only measuring awareness at the end. An individual who discovers the optimal strategy in the final block of the experiment will have a lower optimality rate than their awareness score, even if their initial use of a suboptimal strategy was solely governed by their level of awareness. In an extreme scenario, participants might discover the optimal strategy precisely when they receive the awareness check, suggesting that the awareness check itself could make them aware of the optimal search strategy. Consequently, the awareness score might not accurately reflect their search behaviors during the experiment. More importantly, the current version of the awareness check lacked temporal information regarding when participants were aware of such an optimal strategy (see Lin & Leber, 2024). Nevertheless, taken together, it appears that even when participants know the optimal strategy, they persist in avoiding it, which we interpret to be guided by factors such as effort avoidance (Irons & Leber, 2018).

One might question why we found a more robust effect of preview in the Control group of Experiment 3A than in Experiment 1, in which the preview conditions between the two experiments were essentially the same, albeit with the distinction of grey square placeholders in Experiment 3A. It is possible that the inconsistent results can be solely attributed to the increased sample size. To address this, we conducted a data simulation for the Control group of Experiment 3A (see Online Supplemental Material (OSM) Point 6). The simulation consistently demonstrated a significant preview effect, even with a smaller sample size (N = 24), occurring in more than 99% of instances. Recall that the effect of preview in Experiment 1 was not significant but did have a small effect size in the predicted direction (p = 0.087, d = 0.37). It is possible that the failure to find a significant result in Experiment 1 is simply due to a Type II error; note that the optimality rate in the no-preview condition of Experiment 1 was quite high compared to the Control group in Experiment 3A (see OSM Fig. 8C).

In conclusion, we have better characterized how appraisal time and optimal strategy knowledge jointly impact attentional strategy choice. These are two among likely several important factors that guide how individuals use their attentional systems in everyday life.

### Supplementary Information

Below is the link to the electronic supplementary material.

Supplementary file1 (DOCX 853 KB)

## Data Availability

All data are available via the Open Science Framework (OSF) for all experiments (https://osf.io/52up6/).

## Footnotes

1 One might question whether perceptual differences between red and blue stimuli could drive a difference in choice between red and blue targets. Based on existing data from our lab, we do not feel this is a significant concern, but we provide a more extensive discussion of this issue in the Online Supplementary Materials (OSM) Point 7.

2 As a secondary analysis planned in our pre-registration, we tested whether the order of blocks (participants receiving preview vs. baseline condition first) impacted the results. We did find a significant three-way interaction between order, preview, and epoch. This appeared to be driven by a preview × epoch interaction in the preview-first group. However, we did not replicate this interaction in later experiments containing larger samples. Results are fully detailed in OSM Point 8.

3 Note that, while the correlation was not significant in the Experiment 2 Control group, we have fairly low power to detect reliable relationships with the reduced sample size.

4 One might question why RT was slightly higher – albeit not significantly so – in the Instruction group, given the greater optimality in this group (albeit not significantly so). We conducted a simulation that demonstrated it is possible to observe a higher RT, even with approximately a 10% increase in optimality, due largely to intersubject variance in search speed (see OSM Point 1).

5 In this study, we primarily investigated the effect of the manipulation on optimality. Additional measures, such as switch rates (percent of trials where individuals chose a different target color than on the previous trial) and target eccentricity (the relative distance of the target from the fixation center) are analyzed and provided in the OSM (see Points 2–5).

## Code availability

Experimental code and the data analysis scripts are available via the Open Science Framework (OSF) for all experiments (https://osf.io/52up6/).
