# Supplementary material for: Joint contributions of preview and task instructions on visual search strategy selection
Source: Atten Percept Psychophys. 2024 Apr 24;86(4):1163–75. doi: 10.3758/s13414-024-02870-1 (PMC11093844; doi:10.3758/s13414-024-02870-1)
Supplement: Supplementary file 1 — Supplementary file1 (DOCX 853 KB) [file 13414_2024_2870_MOESM1_ESM.docx]

**Supplementary Materials**

1. RT and Optimality simulation analysis

While we consistently find a strong negative relationship between optimality and RT in ACVS, it does not imply that a difference in optimality would necessarily indicate a significant difference in average RT when comparing groups, as seen in the non-significant RT comparisons reported in Experiments 2 and 3. This lack of significance is largely attributed to substantial variations in individuals' search speeds. To better understand whether a statistically significant group effect should be observed, we conducted two simulations using both in-lab and online data.

Analysis of in-lab data

We combined the data from multiple in-lab ACVS experiments without a preview. A regression line was fitted between optimality and RT (*r* = -.419, *p* < .001) to precisely quantify the RT advantage for searching the smaller subset. Based on the distribution of optimality rates observed in both instruction and control groups observed from Exp 2, we performed 10000 iterations of random samplings of RT values (with 24 data points in each group) from the fitted regression line. The resultant mean RT differences between groups were then calculated. The histogram of the simulated RT differences is presented in Figure 1B. As we can see, given a 13% difference in optimality between instruction and control groups, a 200 ms RT difference is quite typical. Notably, the 95% highest density interval of this distribution includes even negative differences. Thus, statistical comparisons between group means appear not to be as interpretable as the correlation between optimality and RT across participants from both groups.
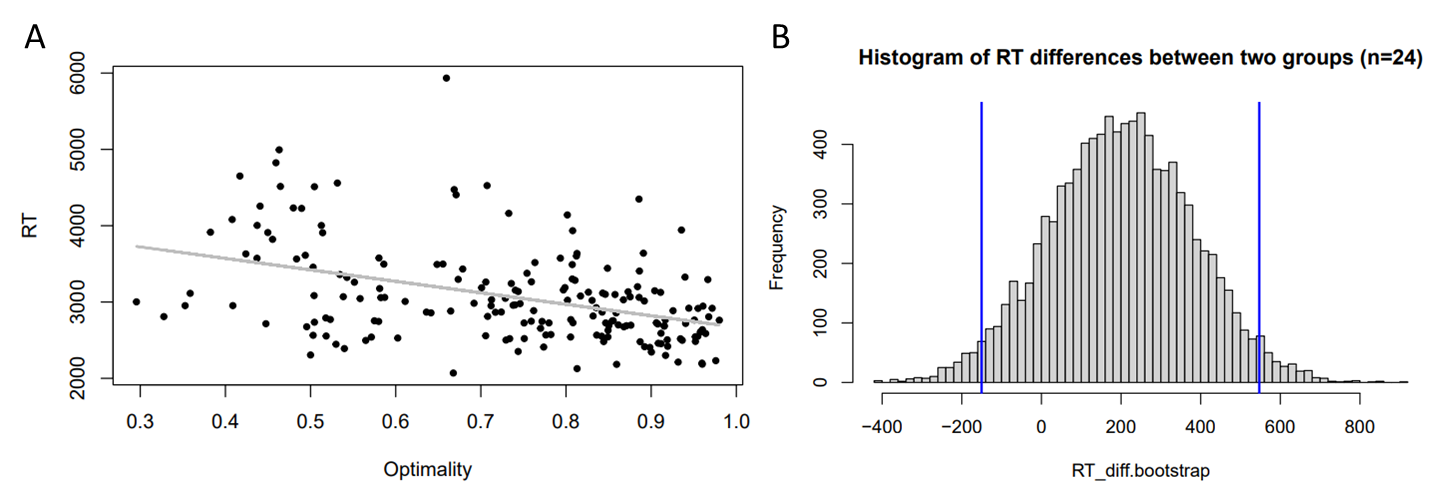


*Supplementary Figure 1*. A) Relationship between optimality and RT. The data were collected in the lab and no preview was included in the ACVS tasks. B) Histogram of the simulated RT differences between two groups with an optimality difference of ~13%.

Analysis of online data

We applied a similar simulation as described above. Here, we combined the data from multiple online experiments, which contained a 1-second preview. Once again, a robust and negative correlation between optimality and RT was observed (*r* = .633, *p* < .001). The simulation results, based on observations from Experiment 3B (with 60 data points in each group and 10,000 iterations), are illustrated in Figure 2B. Notably, the 95% highest density interval of the RT difference between the instruction group and the control group spans from -46.4 ms to 374.5 ms.
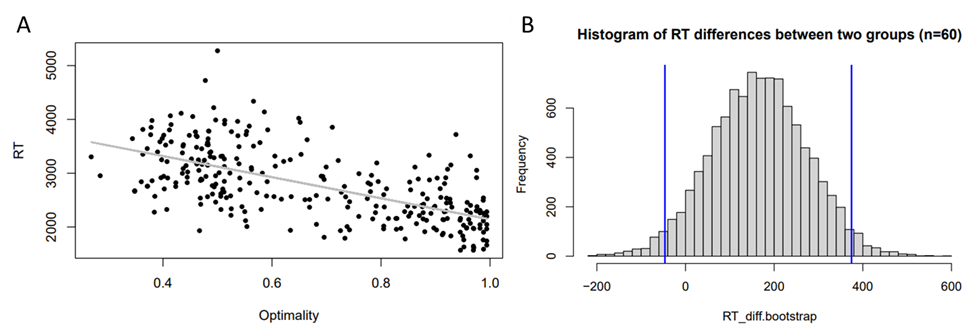


*Supplementary Figure 2*. A) Relationship between optimality and RT. The data were collected online and a preview of 1 second was included in the ACVS tasks. B) Histogram of the simulated RT differences between two groups with an optimality difference of ~11%.

1. Switch rate analysis

We also looked at a secondary measure, switch rate, or the percent of trials in which individuals chose a different target color to the target on the previous trial. While switch rate is to some extent dependent on optimality rate (choose the optimal target on every trial requires switching targets whenever the smallest subset color changes across trials), unnecessary switching often has been associated with slowed RTs in our preview studies (Irons & Leber, 2016; 2018).

The results of switch rates are reported here. In Exp 1, the switch frequency was slightly lower for preview (M = .320) than no-preview (M = .339) conditions (t(23) = 2.225, p = .018, d = .207). In Exp 2, average switch rate did not differ across the two instruction groups (Instruction: M = .318; Control: M = .305, t(46) = 0.502, p = .308, d = .145). In Exp 3A, ANOVA results revealed a main effect of instruction group (F(1,118) = 4.06, p = .045, $\eta_{p}^{2}$ = .033) but not a main effect of preview (F(2,236) = 1.35, p = .260, $\eta_{p}^{2}$= .011). There was no interaction between these two factors, F(2,236) = 0.23, p = .793, $\eta_{p}^{2}$= .002. Means by condition are presented in Table 1. In Exp 3B, ANOVA results did not reveal a main effect for either instruction group (F(1,118) = 0.99, p = .321, $\eta_{p}^{2}$ = .008) or preview (F(2,236) = 0.72, p = .487, $\eta_{p}^{2}$= .006). However, there was an interaction between these two factors, F(2,236) = 3.77, p = .024, $\eta_{p}^{2}$= .031. Means by condition are presented in Table 2. We caution against overinterpreting this finding given the clear lack of a similar interaction in Exp 3A.

Table 1 Switch rates across conditions of preview and instruction from Exp 3A

|  | Preview (ms) | | |
| --- | --- | --- | --- |
| Group | 0 | 1000 | 2000 |
| Instruction | 0.363 | 0.352 | 0.356 |
| Control | 0.396 | 0.389 | 0.384 |

Table 2 Switch rates across conditions of preview and instruction from Exp 3B

|  | Preview (ms) | | |
| --- | --- | --- | --- |
| Group | 250 | 500 | 750 |
| Instruction | 0.351 | 0.350 | 0.358 |
| Control | 0.342 | 0.337 | 0.323 |

1. Impact of the switch of the optimal color on Optimality

We analyzed how the switch of the optimal color impacts optimality in ACVS. Across experiments and conditions, we calculated the optimality for trials of both switch and repeat of the optimal color.

In Exp 1, the switch of the optimal color revealed a significant main effect on optimality (F(1,23) = 14.23, p < .001), in line with previous results (Irons & Leber, 2018, Exp. 2). But, the optimality difference resulting from the switch of the optimal color did not interact with the preview (F(1,23) = 0.38, p = .541; see figure 3A). In Exp 2, the switch of the optimal color again revealed a significant main effect on optimality (F(1,46) = 51.68, p < .001). However, it did not interact with the instruction (F(1,46) = 1.87, p = .178; see figure 3B).


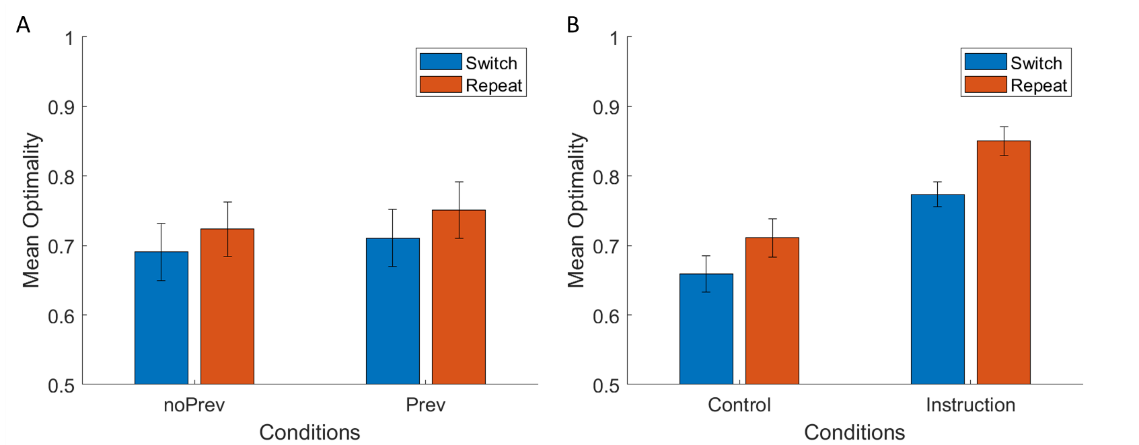


*Supplementary Figure 3*. A) Bar plot of Optimality in trials involving the switch/repeat of the optimal color in Exp 1. B) Bar plot of Optimality in trials involving the switch/repeat of the optimal color in Exp 2.

In Exp 3A, the switch of the optimal color once again revealed a significant main effect on optimality (F(1,118) = 31.92, p < .001). However, the optimality difference resulting from the switch of the optimal color did not interact with either instruction (F(1,118) = 0.69, p = .408) or preview (F(2,236) = 1.60, p = .203; see figure 4 A-C). In Exp 3B, the switch of the optimal color also showed a significant main effect on optimality (F(1,118) = 60.04, p < .001). Furthermore, the optimality difference interacted with the preview (F(2,236) = 5.77, p = .004). Specifically, with longer previews, the difference in optimality between repeat and switch trials decreased (See figure 4 D-F). Given that the same interaction was not significant in Exp. 3A, we caution against overinterpretation of this finding.


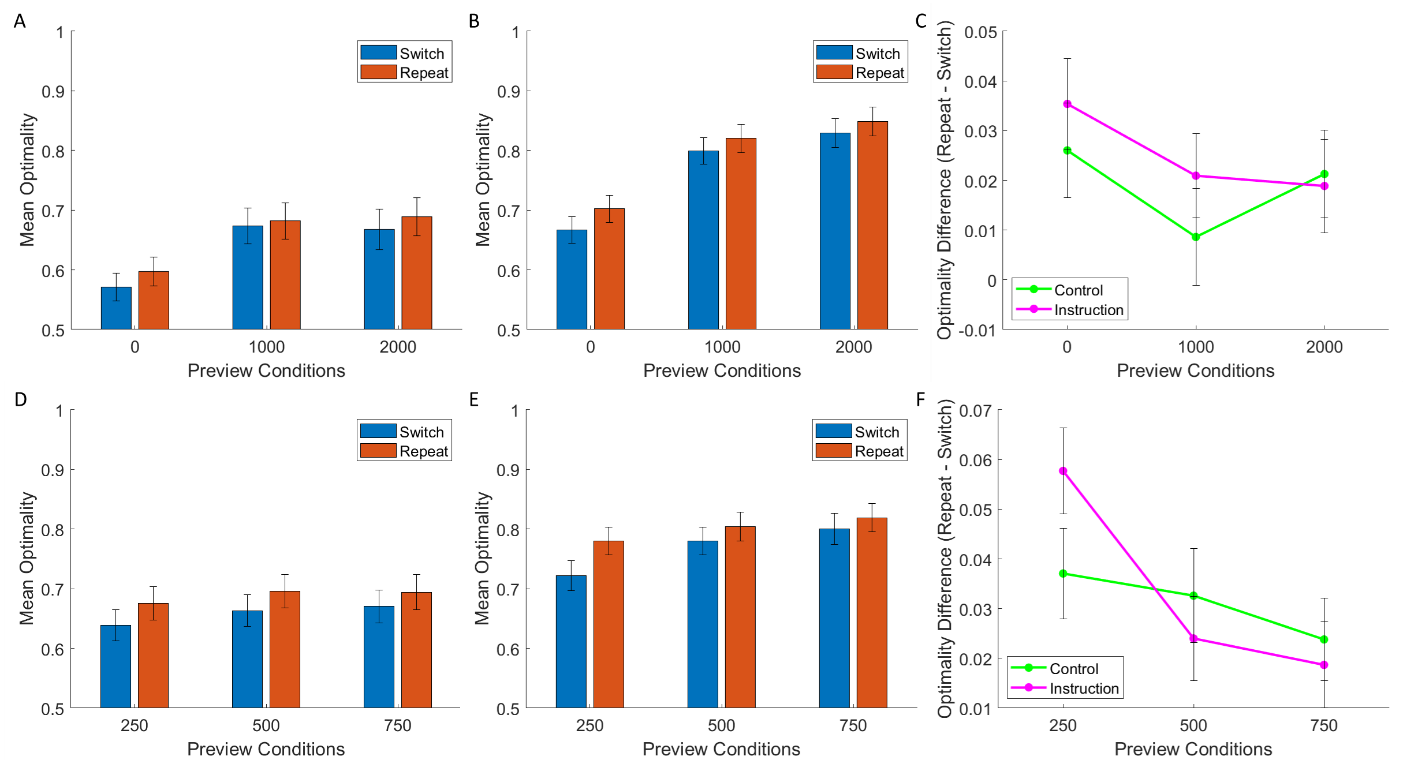


*Supplementary Figure 4*. A) Bar plot of Optimality in trials involving the switch/repeat of the optimal color within the Control group in Exp 3A. B) Bar plot of Optimality in trials involving the switch/repeat of the optimal color within the Instruction group in Exp 3A. C) The optimality difference between the switch/repeat trials across the manipulation conditions in Exp 3A. D) Bar plot of Optimality in trials involving the switch/repeat of the optimal color within the Control group in Exp 3B. E) Bar plot of Optimality in trials involving the switch/repeat of the optimal color within the Instruction group in Exp 3B. F) The optimality difference between the switch/repeat trials across the manipulation conditions in Exp 3B.

1. Relationship between switch rate and optimality

Here, we have depicted scatter plots illustrating the relationship between optimality and switch rate (see Figure 5). The observed suboptimal search behaviors can be predominantly attributed to two factors: excessively frequent switching of the reported target color (~50%, way higher than the rate required for the optimal strategy, ~30%) or a strong tendency to concentrate on one target color without switching.


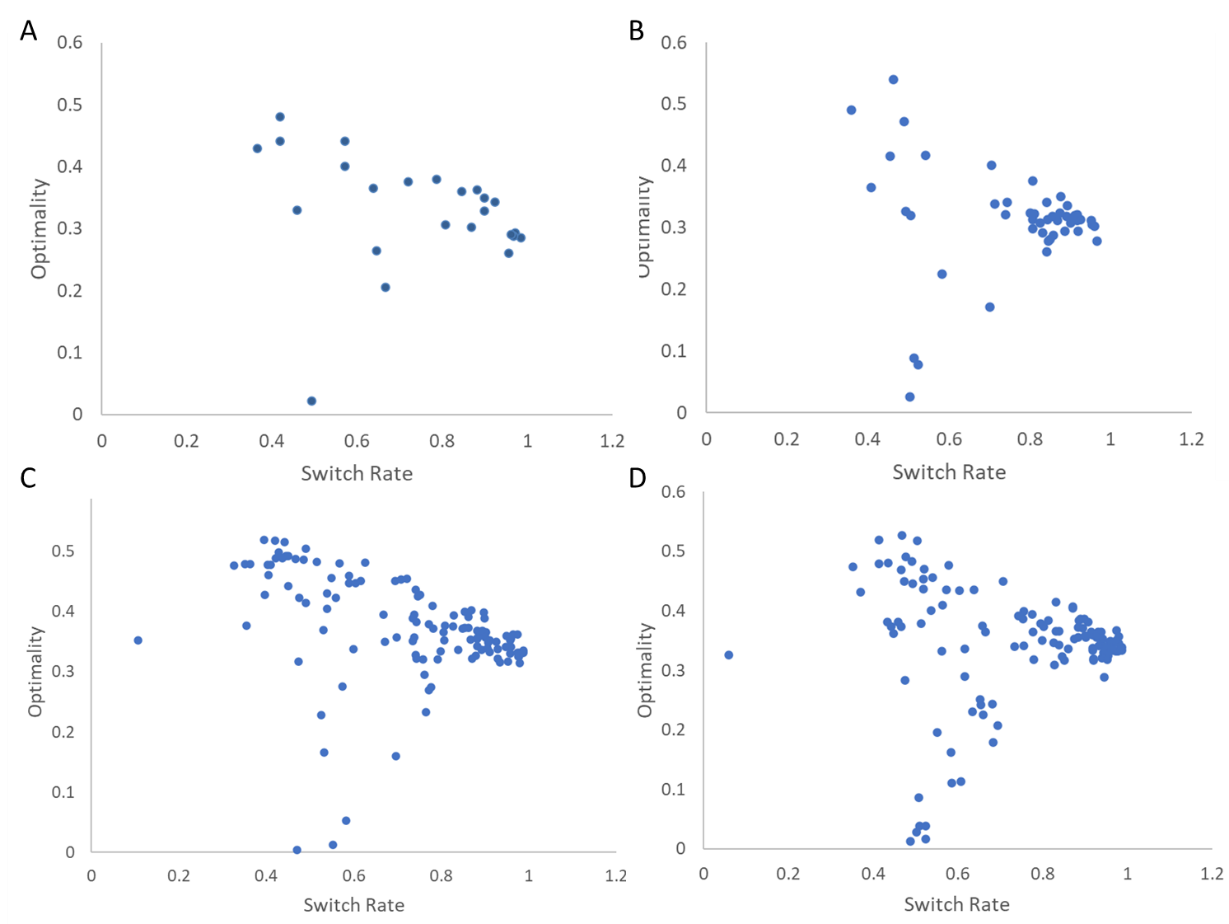


*Supplementary Figure 5*. Scatter plots for Optimality and Switch Rate across all conditions from A) Exp 1;B) Exp 2; C) Exp 3A; D) Exp 3B.

1. Target eccentricity analysis


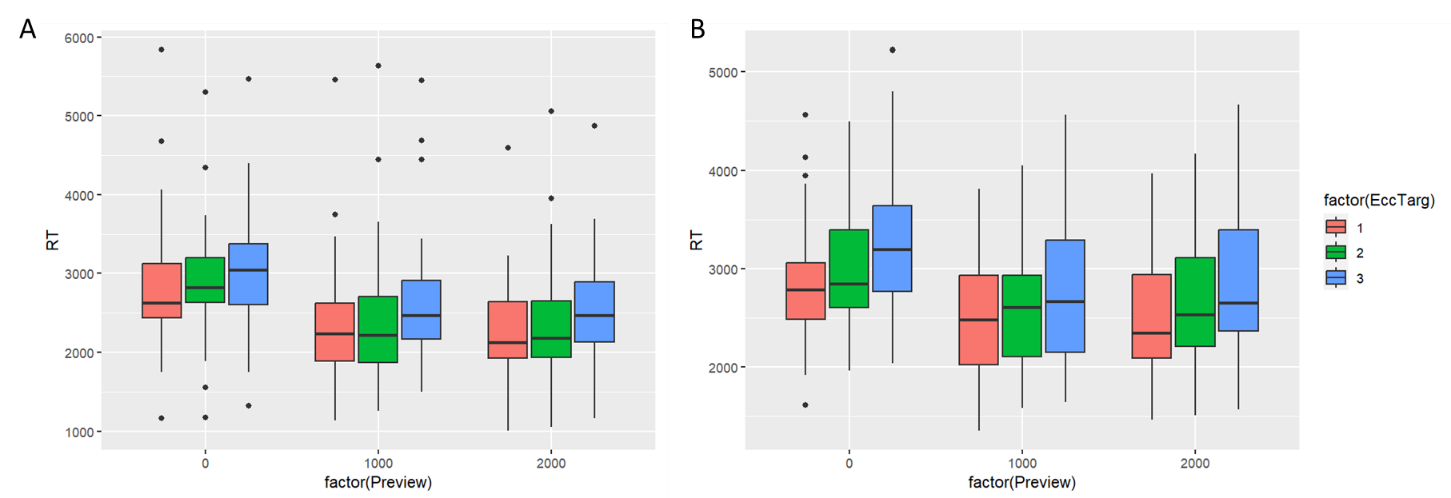


*Supplementary Figure 6*. Boxplots of RTs conditioned by the eccentricity of the target reported and the preview conditions, for A) Instruction group and B) No Instruction group in Exp 3A. Eccentricity value represents the location of the target reported: 1 – inner ring; 2 – middle ring; 3 – outer ring.


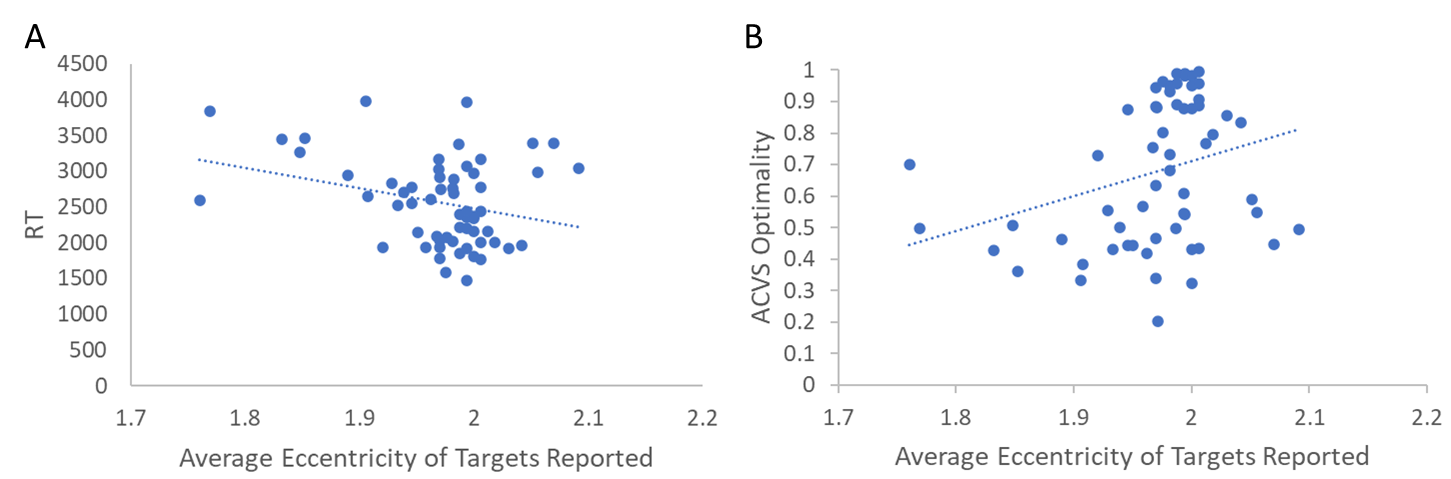


*Supplementary Figure 7.* A. Example of the relationship between RT and average eccentricity of targets reported (No optimal instruction provided with 1 second preview in Exp 3A), as 1 means inner ring, 2 means middle ring, and 3 means outer ring. B. Relationship between ACVS Optimality and average eccentricity of targets reported (No optimal instruction provided with 1 second preview in Exp 3A).

1. Optimality simulation regarding Preview manipulation

Considering that the experimental manipulation of preview in the Control group in Experiment 3A closely resembled that of Experiment 1, one might question why we observed a significant preview effect in Experiment 3A but not in Experiment 1. We ran a simulation to address whether the sample size differences explained the discrepancy. We randomly drew 24 sample participants from the Control group in Exp 3A with replacement and calculated the mean optimality differences between the 1000 ms preview and no preview (0 ms). This process was repeated 10000 times. The histogram of the simulated optimality differences is presented in Figure 8A. The optimality difference we observed in Exp 1 appeared to fall outside the 95% confidence range. The simulation indicates a significant preview effect even with a smaller sample size (N = 24). It is possible that the results of Experiment 1 represented a Type 1 error (failing to reject the null hypothesis when the alternative hypothesis is true). We note that the baseline optimality, measured in the no-preview condition in Exp 1, was quite high compared to our observations from Exp 3A (see Figure 8C). This high baseline optimality might have obscured any potential improvement afforded by the preview.


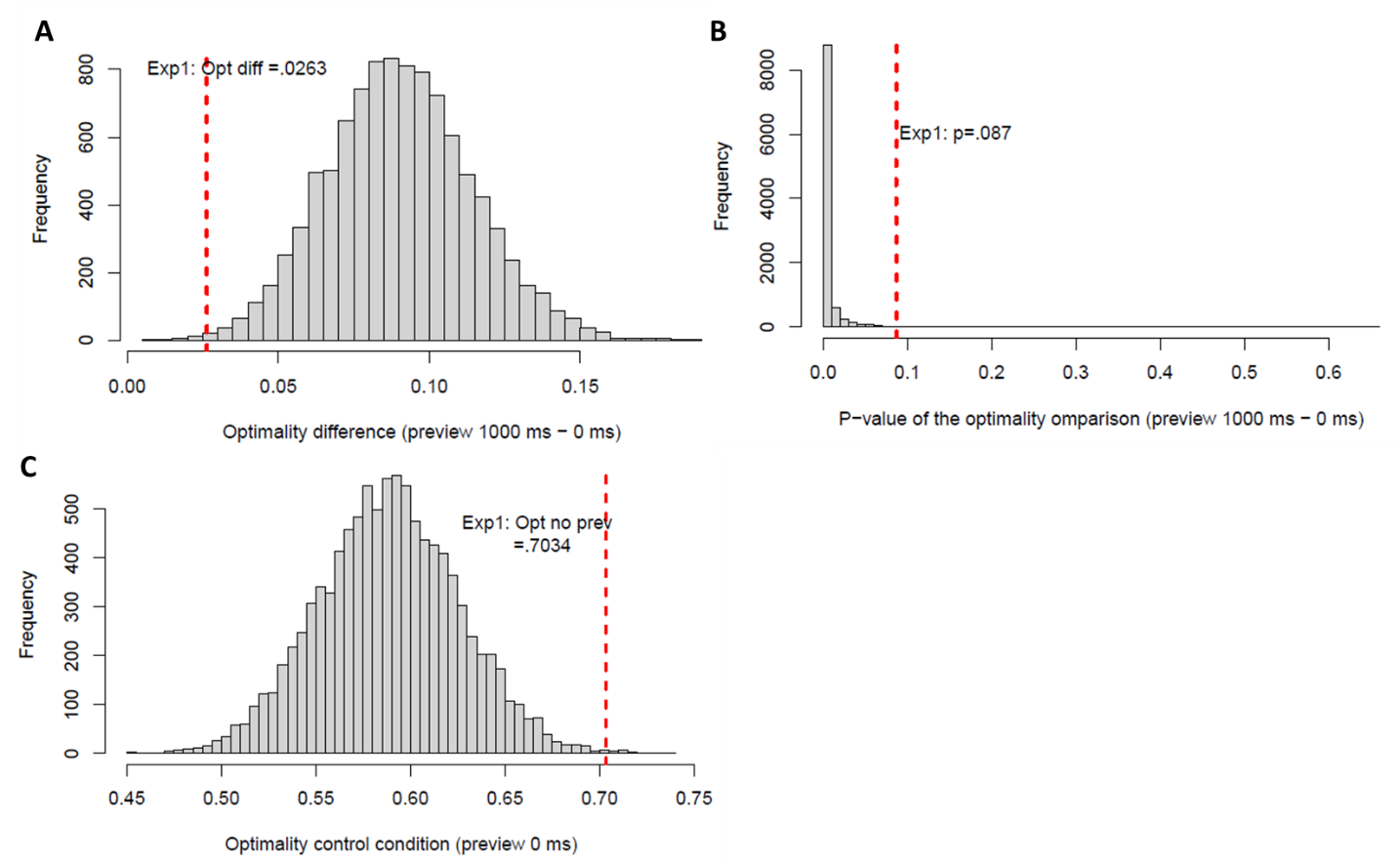


*Supplementary Figure 8.* A. Histogram of the simulated optimality differences between the 1000 ms preview condition and no preview condition (0 ms); Red dashed vertical line represented the value we observed in Exp 1. B. Histogram of the p-values of the simulated optimality comparisons using paired t-test. C. Histogram of the simulated optimality in the no preview condition (0 ms; control condition). Red dashed vertical line represented the value we observed in Exp 1

1. Impact of target square color on RTs

To explore if the target square color impacts people’s reaction time, we compared RT of red targets versus blue targets using data from Exp 3a and 3b, which have the largest sample sizes and, thus, greatest power. We used paired t-tests and did find significant differences in RT in Exp 3a, t(119) = 2.63, p = .009 (Blue M = 2578 ms; Red M = 2666 ms). Exp 3b was similar, t(119) = 2.74, p = .007 (Blue M = 2585 ms; Red M = 2675 ms). While we found significant differences, unlike in our previous analysis on controlled stimulus presentation, we still strongly caution against interpreting these results to mean there were perceptual differences between blue and red. For example, it is possible that our participants had aesthetic preferences for blue over red stimuli. We checked whether people chose blue more often than red and found a slight numerical preference, which was not significant. Specifically, participants chose the blue target on 51.7% of trials in Exp 3a and 51.5% of trials in Exp 3b. We compared the total choice preference for blue vs. red per participant vs. a hypothesized mean difference of 0, and the results were not significant in Experiment 3a, t(119) = 1.609, p = 0.110, or in Experiment 3b, t(119) = 1.181, p = 0.239. Critically, these results show that the ~90-ms faster RT for blue vs. red trials did not significantly drive a preference for choosing blue targets. Thus, even if the RT difference were driven by perceptual differences, one cannot argue that suboptimal behavior is driven by an overall bias to search for blue targets.

Here, the main point we must emphasize is that even if a true 90-ms perceptual advantage for searching blue vs. red exists, it cannot provide a rational explanation for why someone might choose a suboptimal vs. optimal strategy, since we can estimate the advantage for optimal vs. suboptimal strategies to be considerably larger (e.g., based on the regression line of optimality vs. RT in for online data (see supplementary material point 1), we calculate a 981 ms advantage). All things considered, we accept that RT differences might emerge between red and blue in the ACVS, even when they do not show up in our controlled cued-color tasks. However, they do not provide an explanation for the optimality of people’s strategy.
